# Preheated (Heat-Assisted) Clinching Process for Al/CFRP Cross-Tension Specimens

**DOI:** 10.3390/ma13184170

**Published:** 2020-09-19

**Authors:** Pai-Chen Lin, Jun-Chang Fang, Jia-Wei Lin, Xuan Van Tran, Yern-Chee Ching

**Affiliations:** 1 Advanced Institute of Manufacturing with High-tech Innovations & Department of Mechanical Engineering, National Chung-Cheng University, Chiayi 62102, Taiwan; gavin60506058311@gmail.com (J.-C.F.); peterx8888x@yahoo.com.tw (J.-W.L.); 2 Institute of Development of Strategy, Thu Dau Mot University, Phu Hoa Ward, Thu Dau Mot City 0650, Vietnam; xuantv@tdmu.edu.vn; 3 Department of Chemical Engineering, University of Malaya, Kuala Lumpur 50603, Malaysia; chingyc@um.edu.my

**Keywords:** clinching, joining composite, modifications of clinching joining technologies, carbon-fiber-reinforced-plastic, aluminum alloy, fatigue

## Abstract

Effects of processing parameters on preheated (heat-assisted) clinching process to join aluminum alloy 5052-H32 (AA5052) and thermoplastic carbon-fiber-reinforced-plastic (TP-CFRP) sheets for cross-tension (CT) specimens were first studied. Preheating was critical since brittle TP-CFRP could be softened to avoid fracturing or cracking during clinching process. Four processing parameters, including punching force, die depth, heating mode, and heating temperature, were considered. Quasi-static tests and microscope observations were taken to evaluate AA5052/TP-CFRP clinch joints in CT specimens and determine appropriate processing parameters for fatigue tests. Finally, fatigue data and failure mode of clinch joints in CT specimens were obtained and discussed.

## 1. Introduction

The greenhouse gases, such as carbon oxide, emitted from vehicles have significant contributions on the global warming and climate change. Therefore, reducing emissions of greenhouse gases by manufacturing lightweight vehicles with safe structures and low costs becomes a difficult but inevitable goal of the automotive industry. In order to achieve this goal, recently, the concept of “right-weight” design is adopted by automotive companies to optimize the weight, strength, and cost of their car bodies simultaneously [1,2]. Based on this concept, various types of advanced lightweight materials are used to make hybrid or multi-material car bodies, e.g., aluminum alloys (Al), carbon-fiber-reinforced-plastic (CFRP), and high strength steels (Fe).

Despite the advantages brought by using multiple materials in car bodies, joining dissimilar materials, e.g., Al-to-CFRP (Al/CFRP) and Al-to-Fe (Al/Fe), always gives manufacturing engineers lots of challenges due to their distinct physical and chemical properties. For dissimilar joining between Al and Fe sheets, the automotive industry prefers using mechanical fastening processes to using conventional resistance spot welding (RSW) to make joints. The production lines currently apply flow drill screwing (FDS) [3], self-piercing riveting (SPR) [4,5], friction stir spot welding (FSSW) [6,7,8], clinching [9,10], etc. to make Al/Fe joints in hybrid car bodies. For dissimilar joining between Al and CFRP sheets, many advanced joining processes have been proposed in recent years, including adhesive bonding, diffusion bonding, SPR [11,12], FDS [13], bolted joints, clinching [14,15,16,17,18,19,20,21,22,23,24,25,26,27], solid-state welding [28,29,30,31,32,33], and laser assisted welding [34,35], as reported in Pramanik et al. [36]. Among these processes, BMW AG currently selected SPR, FDS, bolted joining, and adhesive bonding to make Al/CFRP joints in hybrid car bodies for electric vehicles and luxury sedans in the production lines [37].

Although the strength and reliability of above joining processes are quite good, several shortcomings of them are inevitable, for example, the curing time of adhesive bonding, the degradation of adhesive bonding strength under ultraviolet light, and the additional weight and cost of fasteners for SPR, FDS, and bolted joining. Therefore, the clinching process is considered as an alternative since it has many unique advantages over the above processes, such as comparable mechanical performance, no additional weight, short processing time, and low purchase and operation cost. Note that the clinching process is a quite old technology, which was first patented in Germany in 1897 (DRP-NR. 98517). Although this process has been developed for a long time, it started to be used to join automotive components and car bodies since 1980s. The main reason is the increasing use of several advanced lightweight materials, e.g., Al and CFRP, which are impossible or difficult to be joined by the conventional RSW process.

Lambiase and Ko [21] used four punches with different diameters and taper angles to make clinch joints between Al and thermoset CFRP (TS-CFRP) at the room temperature. They reported that the joints made by the punch with larger diameter and smaller taper angle have better mechanical performance. It should be noted that the lower button (TS-CFRP) of the joint showed many cracks and fractures near its bottom side. Lambiase et al. [22] used four types of dies, round split, round grooved, round flat and rectangular, to make dissimilar clinch joints between Al sheets and glass fiber reinforced plastic (GFRP) sheets at the room temperature. Among four types of dies, the round split die provided the best mechanical interlock and mechanical performance. Note that similar defects can be seen in the lower button (GFRP) of the joints as well. According to the above research works [21,22], the lack of ductility for GFRP and TS-CFRP at the room temperature is one possible reason for the defects or failures of joints.

To overcome the challenge from brittle materials, such as polymer and composite materials, Lambiase [18,19] and Lambiase and Ilio [20] developed a preheated (heat-assisted) clinching process to join aluminum and thermoplastic polymer sheets. In this process, the heater gun was used to blow 200–400 °C air flow to heat the thermoplastic polymer sheets for certain time before clinching. Lin et al. [25,26] adopted this concept to join aluminum alloy 5052-H32 (AA5052) and thermoplastic CFRP (TP-CFRP) sheets. In their process, the heat plate was used to heat TP-CFRP sheets at 100 °C to improve their ductility. It is quite interesting that the dissimilar clinch joints reported above only show relatively small defects in their lower buttons (TP-polymer or TP-CFRP).

Many researchers also consider different ways to modify the clinching process for making metal-to-composite joints. Huang et al. [14] used preheating process, dummy sheet, and adhesive bonding to improve the clinching process for joining AA2017 and TS-CFRP sheets. Only small delamination could be seen inside the AA2017/TS-CFRP clinch joint. Lee et al. [15,16,17] adopted the concept of riveting to develop the hole-clinching process and make Al/Fe, Al/TS-CFRP, and Fe/TS-CFRP clinch joints. Due to the pre-drilling process, the defect problem of TS-CFRP sheets could be avoided. Lambiase and Ko [23] added the reshaping process (secondary step) after the clinching process (first step) to improve the mechanical interlock and joint strength of Al/TS-CFRP clinch joints. Note that the clinch joints made by the two-step clinching process had similar geometries as those made by the hole-clinching process since their bottom portions (TS-CFRP) were severely fractured. Lambiase and Paoletti [24] used rotating punch to apply frictional heat and punching force simultaneously during the clinching process of aluminum and CFRP sheets, called friction-assisted clinching process. In this process, the CFRP sheets were predrilled to avoid severe defects. The aluminum sheets were heated and softened by frictional heat to avoid fracture during the clinching process.

As mentioned earlier, Lin et al. [25,26] studied the effects of processing parameters, such as punching force, die geometry, and punch geometry, on the preheated clinching process of AA5052 and TP-CFRP sheets for lap-shear (LS) specimens experimentally. Appropriate processing parameters were obtained. The mechanical performance of AA5052/TP-CFRP clinch joints subjected to shear dominant loads was obtained. However, that of AA5052/TP-CFRP clinch joints subjected to opening dominant loads, which are also important in the practical applications, has not been studied. In addition, the processing parameters for LS specimens may not be suitable for cross-tension (CT) specimens since their sizes and loading conditions are quite different. Therefore, in this study, the effects of processing parameters on the preheated (heat-assisted) clinching process of AA5052 and TP-CFRP sheets for CT specimens were first studied. Four important parameters, including heating mode, punching force, heating temperature, and die geometry, were considered. Note that the preheating is particularly studied here since the specimen size may significantly affect the heat conduction and specimen temperature during the clinching process. Quasi-static tensile tests, fatigue tensile tests, and microscopic observations are then conducted to evaluate the mechanical performance of AA5052/TP-CFRP dissimilar clinch joints in CT specimens. An appropriate setup for clinch joints in CT specimens is then obtained to conduct following fatigue tests. Fatigue data and failure modes of clinch joints in CT specimens are obtained and discussed.

## 2. Materials and Methods

### 2.1. Materials

In order to make dissimilar clinch joints, 1.6 mm thick AA5052-H32 and TP-CFRP sheets were taken. Their mechanical properties are listed in Table 1.

**Figure 1:**
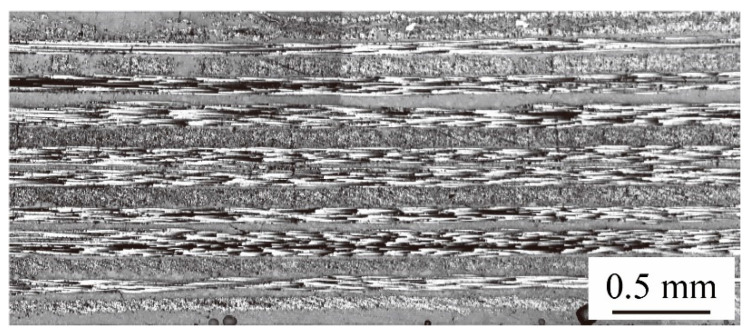
A cross section of a 1.6 mm thick thermoplastic carbon-fiber-reinforced-plastic (TP-CFRP) sheet from TOPKEY Inc. [26].

Figure 1 shows the microstructure of a 1.6 mm thick TP-CFRP sheet from TOPKEY Inc. (Taichung, Taiwan).

The TP-CFRP sheet has sixteen cross-layup layers. Each layup layer has a thickness of 0.1 mm. The orientations of the layers are given as [0°/90°/90°/0°/0°/90°/90°/0°/0°/90°/90°/0°/0°/90°/90°/0°]. The matrix of TP-CFRP is made of polycarbonate (PC) with the glass transition temperature (Tg) of 140 °C [38].

### 2.2. Preheated (Heat-Assisted) Clinching Process

**Figure 2:**
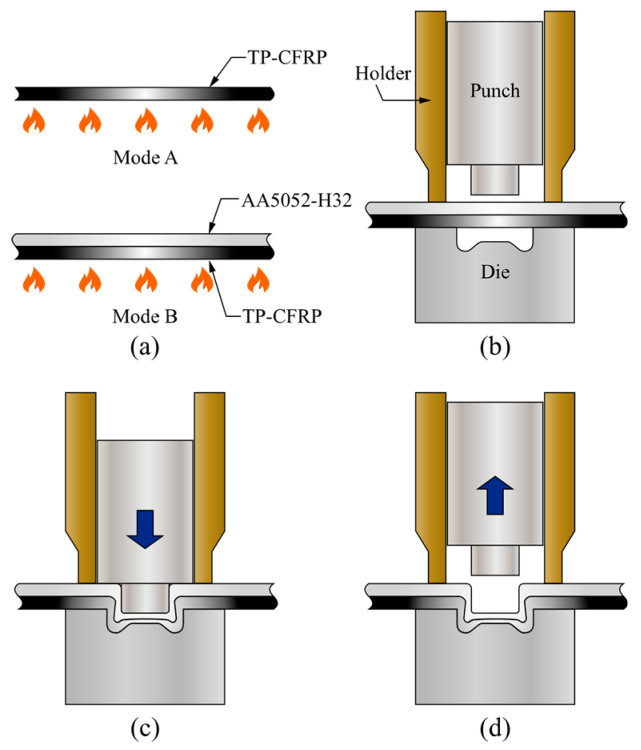
Preheated (heat-assisted) clinching process for AA5052/TP-CFRP dissimilar clinch joints: (**a**) Preheating, (**b**) clamping, (**c**) punching, (**d**) retracting.

The traditional clinching process is a simple sheet metal forming method in which a punch directly pushes similar or dissimilar sheet metals into a die without any additional fastener. The preheated (heat-assisted) clinching process is a modified one for joining AA5052 and TP-CFRP sheets, as shown in Figure 2 A TP-CFRP sheet (Mode A) or both AA5052 and TP-CFRP sheets (Mode B) are heated before clinching by a heat plate, as shown in Figure 2a.

But the die, holder, and punch shown in Figure 2b are not preheated before clinching. After preheating, the AA5052 sheet (upper) and the TP-CFRP sheet (lower) are held by the holder and die, as shown in Figure 2b. Then the punch pushes the two sheets into the die to plastically deform them as two buttons. A strong mechanical interlock between them are formed as the punch continuously pushes to a certain level, as shown in Figure 2c. The mechanical interlock holds the two sheets tightly together. Finally, an AA5052/TP-CFRP dissimilar clinch joint is made after the punch is released, as shown in Figure 2d.

Note that in Figure 2a, the heating temperatures were measured by an infrared thermometer. According to the manual, its accuracy and repeatability are ±2 °C and ±1 °C, respectively. For each case, three measurements were conducted at the specimen center within an area of 1 *in*^2^. The time for clamping and punching, shown in Figure 2b,c, in general is less than 1 s. and the thermal conductivity of PC coating on the TP-CFRP sheet is much lower than metals. Therefore, the heat loss in these two stages should be small. In Figure 2d, the joint should be cooled down to the room temperature before retracting from the die to avoid damaging the interlock.

### 2.3. Punch and Die Designs

**Figure 3:**
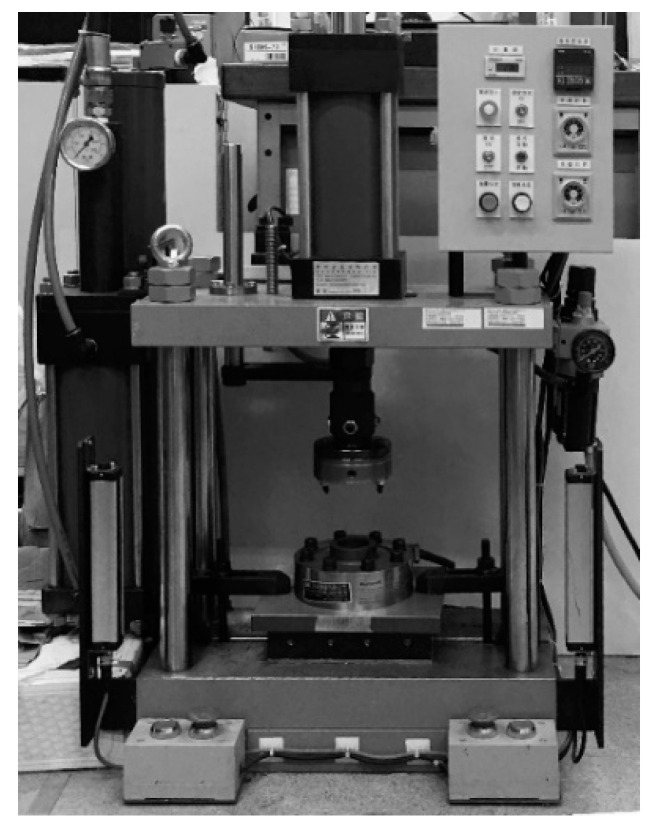
A hydro-pneumatic press machine, ARC-10 T, made by APMATIC Corp, Taiwan [26].

In this study, the clinching process is conducted by a punch and a die installed in a hydro-pneumatic press machine (APMATIC Corp, New Taipei, Taiwan) in Figure 3.

**Figure 4:**
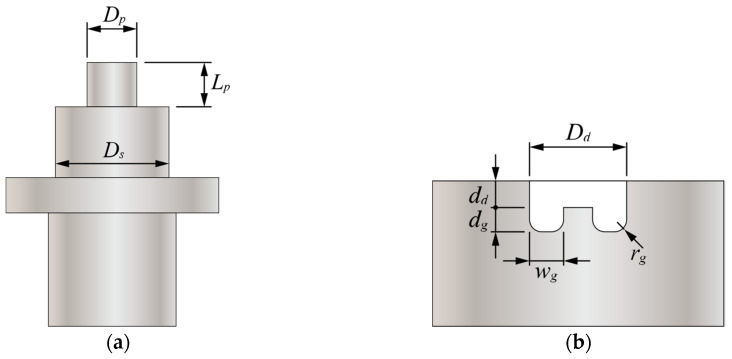
Designs of (**a**) a circular punch and (**b**) a circular die with a groove.

A 22 kN load cell was installed under the die to obtain accurate punching forces. Figure 4 shows the designs of the circular punch and die.

The dimensions of one circular punch, P1, and two circular dies, D1 and D2, used in this study are listed in Table 2.

The $D_{p}$ and $L_{p}$ represent the diameter and length of the punch probe, respectively. The $D_{s}$ represents the diameter of the punch shoulder. The $D_{d}$ and $d_{d}$ represent the diameter and depth of the die, respectively. The $w_{g}$, $r_{g}$, and $d_{g}$ represent the width, radius, and depth of the die groove, respectively.

### 2.4. Specimen Design

**Figure 5:**
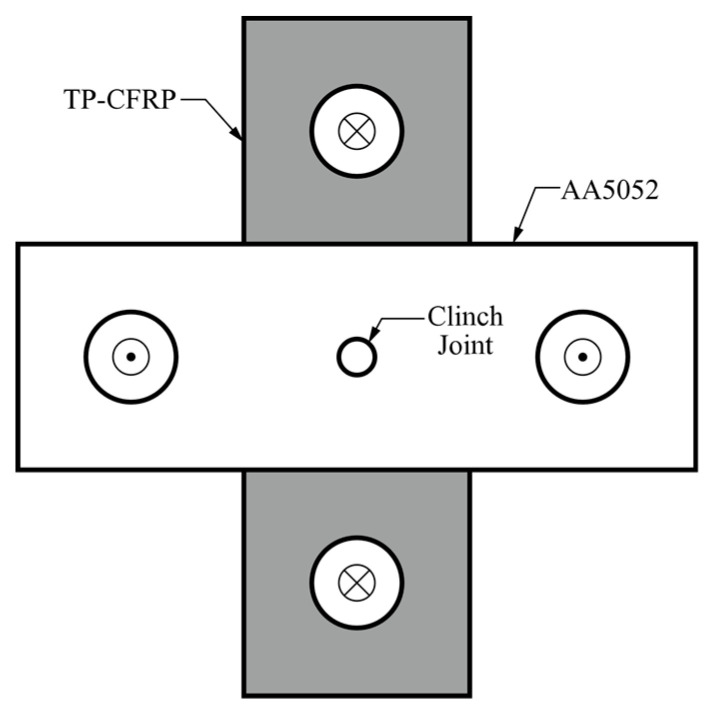
A cross-tension (CT) specimen with a clinch joint made of AA5052-H32 and TP-CFRP sheets.

According to the AWS C1.1M/C1.1:2000 standard [39], CT specimens are used to evaluate the mechanical performance of clinch joints subjected to opening dominant loads shown in Figure 5.

The white plate is made of AA5052-H32 and the gray plate is made of TP-CFRP. Both plates have 50.8 mm width and 152.4 mm length. Two circular holes located at the ends of each plate have 20 mm diameter. The overlap region at the specimen center has 50.8 mm width and 50.8 mm length. The cross and dot symbols marked at the center of four circular holes indicate the applied load directions.

### 2.5. Quasi-Static and Fatigue Tests

Quasi-static tensile tests were conducted by applying monotonic displacements at a rate of 0.07 mm/s until failure by a hydraulic testing machine made by MTS Systems Corp. Load-displacement curves and failure modes were recorded. Three to five replicates were conducted to obtain average failure loads. The average failure load was then taken as a reference load for the following fatigue tests. Fatigue tests were conducted by applying cyclic loads with load ratio R of 0.1, waveform of sinusoid, and testing frequency of 2 Hz to each CT specimen until failure by the same machine. Fatigue lives and failure modes were recorded. Three to five replicates per load range were conducted to obtain corresponding fatigue data. After fatigue tests, the failed specimens were cross sectioned and observed by optical microscopes to study their fatigue behavior and failure mechanisms. Note that the quasi-static and fatigue tests are both designed based on the AWS C1.1M/C1.1:2000 standard [38]. The testing protocol of the quasi-static and fatigue tests are listed in Table 3.

## 3. AA5052/TP-CFRP Clinch Joint

**Figure 6:**
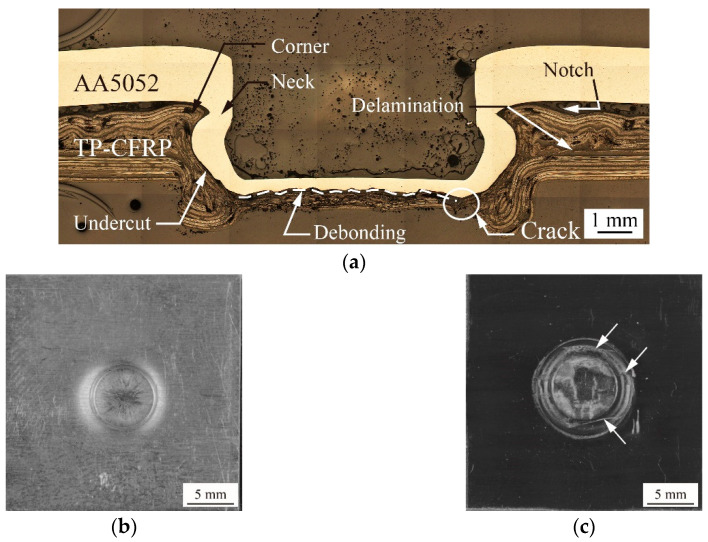
(**a**) A cross-sectional micrograph of an AA5052/TP-CFRP dissimilar clinch joint. (**b**) The top and (**c**) the bottom views of another AA5052/TP-CFRP dissimilar clinch joint.

The detailed geometry of an AA5052/TP-CFRP dissimilar clinch joint can be seen in a cross-sectional micrograph in Figure 6a.

The Punch P1, Die D1, punching force of 69 kN, Heating Mode B, and heating temperature of 100 °C were used to make this joint. In Figure 6a, the clinch joint has a good mechanical interlock which consists of a thin neck (marked by an arrow) and a deep undercut (marked by an arrow). It should be noted that no defect can be seen in the inner button (AA5052); however, several tiny defects (marked by arrows) can be seen at the corner and bottom of the outer button (TP-CFRP) where large plastic deformation occurs during clinching. In Figure 6b, a circular hole is indented into the top surface of the joint. In Figure 6c, a circular cylinder rounded by a ring is protruded from the bottom surface of the joint. Several thin cracks (marked by arrows) on the ring can be found. As reported in Lin et al. [25,26], the aforementioned defects in general have minor effects on the mechanical interlock and failure load since the failure and fatigue cracks usually occur away from those regions with defects.

## 4. Results and Discussion

According to the research works of Lin et al. [25,26], the appropriate processing parameters for AA5052/TP-CFRP clinch joints in LS specimens are given as the punching force of 61 kN, Heating Mode A, and heating temperature of 100 °C. However, the size and shape of CT specimens are quite different to that of LS specimens. The processing parameters for LS specimens may not be suitable for CT specimens. Therefore, a preliminary study of the heating mode effects on the clinching process for CT specimens is first conducted. Then a study of the heating temperature effects and die depth effects for CT specimens is conducted. Once the appropriate setup for CT specimens is determined, a fatigue test is conducted to further understand their fatigue performance.

### 4.1. Heating Mode Effects

The main objective of this section is to select an appropriate heating mode for CT specimens. In order to further understand the effects of heating mode on the deformation of AA5052 and TP-CFRP sheets, various punching forces are considered as well. In this section, two types of heating modes, Heating Modes A and B shown in Figure 2a, and five punching forces, including 66, 69, 70, 73, and 75 kN, are studied together. Note that the spacings between different punching forces are not equal since the press machine used here is a basic model without closed loop control. The accurate punching force is measured by one additional load cell (Model 41, Honeywell).

**Figure 7:**
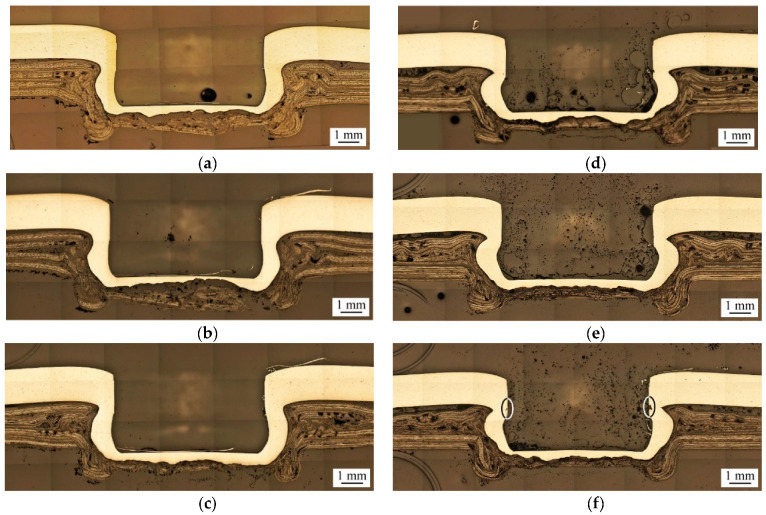
Cross-sectional micrographs of clinch joints made under Heating Mode A with (**a**) 69 kN, (**b**) 70 kN, (**c**) 75 kN punching forces, and Heating Mode B with (**d**) 69 kN, (**e**) 70 kN, (**f**) 75 kN punching Figure 1. Die D1 and heating temperature of 100 °C are used.

Figure 7 shows the effects of heating modes and punching forces on the geometry of clinch joints.

Two heating modes, A and B, and three punching forces, 69, 70, and 75 kN, are considered. As mentioned in Figure 2a, Heating Mode A means that only the TP-CFRP sheet is heated, while Heating Mode B means that both AA5052 and TP-CFRP sheets are heated simultaneously. The heating temperature is selected as 100 °C. After preheating, the clinch joints are made by Punch P1 and die D1, as listed in Table 2.

Figure 7a–c show clinch joints made under Heating Mode A with 69 kN, 70 kN, and 75 kN punching forces, respectively. In these figures, when the punching force increases, the interlock of clinch joints becomes stronger since the undercut size gradually increases, and the neck thickness gradually decreases. Figure 7d–f show clinch joints made under Heating Mode B with 69 kN, 70 kN, and 75 kN punching forces, respectively. In these figures, when the punching force increases, these clinch joints have a similar trend of joint geometry as those made under Heating Mode A. However, the clinch joint made under 75 kN punching force has tiny cracks near its necks, as marked by two ellipses in Figure 7f. The tiny cracks may damage the joint strength significantly. Comparing with the clinch joints made under Heating Mode A, the clinch joints made under Heating Mode B have thinner neck thicknesses and larger undercut sizes probably because the preheating process softens the AA5052 and TP-CFRP sheets simultaneously, and then the punch deforms them more easily.

**Figure 8:**
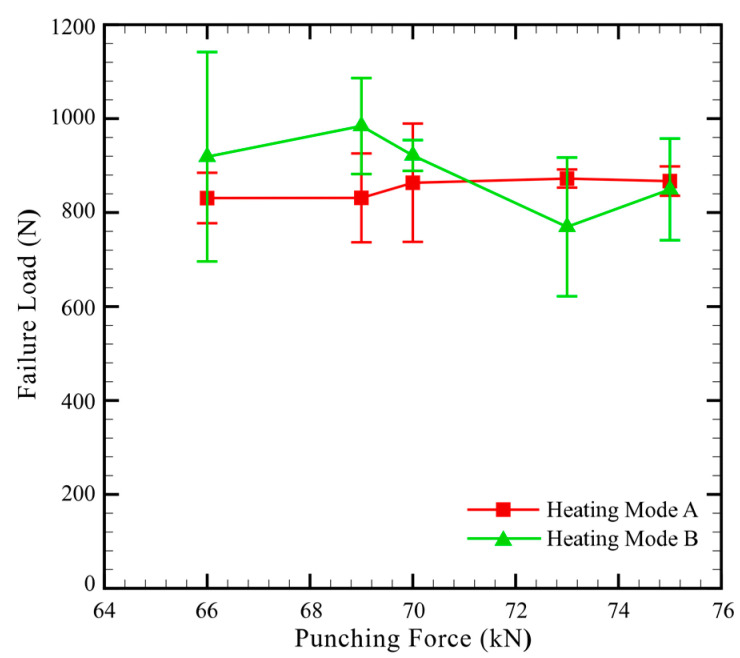
Failure load vs. punching force of dissimilar clinch joints in CT specimens made under Heating Modes A and B. The Punch P1, Die D1 and heating temperature of 100 °C are used.

Figure 8 plots the effects of heating modes and punching forces on the failure loads of clinch joints in CT specimens.

As shown in Figure 8, under Heating Mode A, the clinch joints made under punching forces from 66 to 75 kN have nearly constant failure loads. The maximum failure load occurs at the punching force of 73 kN. This is probably because its mechanical interlock is slightly stronger than those of the other clinch joints as shown in Figure 7. In contrast to the clinch joints made under Heating Mode A, those made under Heating Mode B and punching forces from 66 to 70 kN have higher failure loads. This is probably because the clinch joints in Figure 7d,e have better interlocks than those in Figure 7a,b. However, the clinch joints made under Heating Mode B and punching forces from 70 to 75 kN have lower failure loads. This is probably because those clinch joints have some cracks near their necks, as shown in Figure 7f which may weaken the interlocks and failure loads. In addition, the corner structures near the necks of the clinch joint in Figure 7f are thinner than those in Figure 7c. This fact may weaken the interlocks and failure loads as well, especially under opening loading conditions.

As shown in Figure 8, the standard deviations of some results are quite high. One possible reason is the number of replicates. However, for the results with higher standard deviations, five replicates were conducted as mentioned in Section 2.5. Therefore, the number of replicates may not be the main reason. Another possible reason is that the interlock of the joint and the defects inside the outer button (TP-CFRP) are not perfectly symmetric, as shown in Figure 7. This fact indicates that the deformations of the inner and lower buttons are slightly non-uniform along their circumferences. As can be seen in quasi-static tensile tests, the failure of the joint and corresponding failure load were dominated by the corner of the lower button and small defects inside it, which are strongly dependent on the deformation of the inner and lower buttons.

From this viewpoint, the high standard deviations shown in Figure 8 may be caused by the slightly non-uniform deformation of the inner and lower buttons.

According to the above discussions, the AA5052/TP-CFRP dissimilar clinch joint made under a punching force of 69 kN and Heating Mode B provides the best interlock and the maximum average failure load among them. Therefore, the punching force of 69 kN and Heating Mode B are adopted together in the following fatigue tests.

### 4.2. Heating Temperature Effects

The objective of this section is to optimize the heating temperature under Heating Mode B to further improve the strength of clinch joints in CT specimens. Therefore, only a small range of heating temperatures (97 to 115 °C) with a relatively small spacing (3 °C) was considered here in order to have appropriate ductility and strength for TP-CFRP and AA5052 sheets at the same time. Note that the heating temperatures selected here are 43 to 25 °C lower than the Tg (140 °C) of PC. The main reason to select relatively low temperatures here is that during the clinching process, the TP-CFRP sheet not only needs to be softened by heating to obtain appropriate ductility but also needs to keep its strength at certain level to avoid being crushed by the punching force and the deformed aluminum sheet. Consequently, the mechanical interlock of the joint can be made.

**Figure 9:**
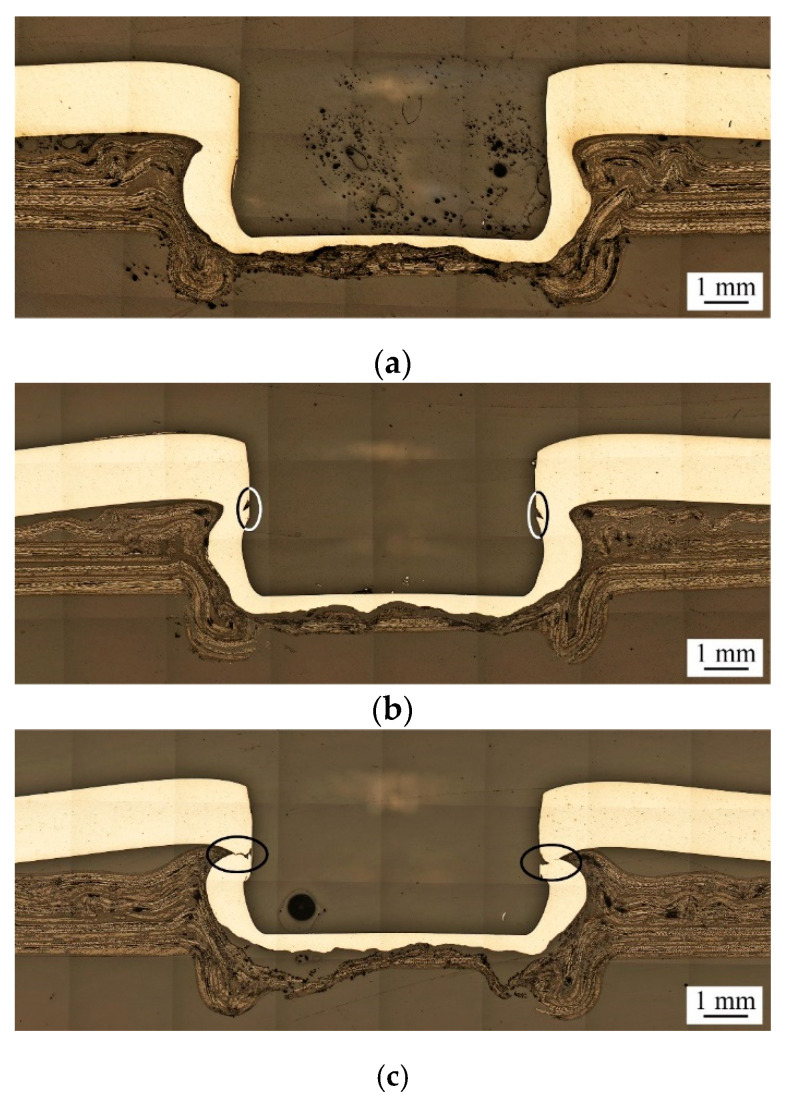
Cross-sectional micrographs of clinch joints under heating temperatures of (**a**) 97 °C, (**b**) 103 °C, and (**c**) 112 °C. The Punch P1, Die D1, punching force of 69 kN, and Heating Mode B are used.

Figure 9 shows the effects of heating temperature on the geometry of clinch joints.

Three heating temperatures, 97 °C, 103 °C and 112 °C, are considered. The clinch joints are made under punching force of 69 kN and Heating Mode B with Punch P1 and Die D1. In Figure 9, when the heating temperature increases, the interlock of clinch joints becomes stronger since the undercut size gradually increases, and the neck thickness gradually decreases. However, when the heating temperature increases above 103 °C, some small and large surface cracks can be found near the neck, as marked by ellipses in Figure 9b,c. The corner structures nearby the necks become thinner as well. This fact indicates that increasing heating temperature can soften the AA5052 and TP-CFRP sheets, increase their plastic deformation, and improve the interlock of the clinch joint. However, it may also cause excessive plastic deformation near some critical locations of the clinch joint, such as the neck and undercut, and finally result in small and large surface cracks there. These small and large surface cracks may become the crack or failure initiation sites to cause joint failures and weaken the corresponding joint strengths.

**Figure 10:**
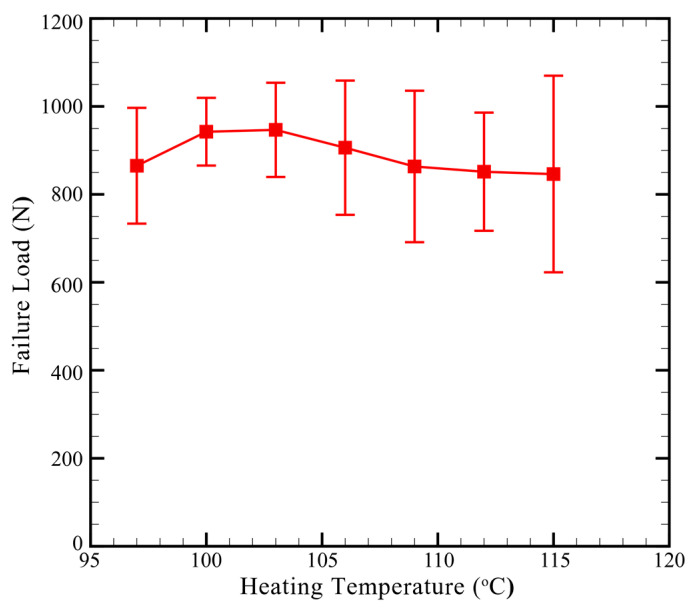
Failure load vs. heating temperature of clinch joints in CT specimens. The Punch P1, Die D1, punching force of 69 kN, and Heating Mode B are used.

Figure 10 plots the effects of heating temperature on the failure load of clinch joints in CT specimens.

In Figure 10, when the heating temperature increases, the failure load gradually increases and then decreases. The failure load achieves the maximum value at the heating temperature of, 103 °C. As discussed in Figure 9, increasing heating temperature not only improves the interlock of clinch joints but also induces small or large cracks near the neck. Some large cracks even grow through the necks. Apparently, the former factor (interlock) improves the joint strength while the latter factor (crack) weakens that. The struggle between opposite effects probably results in the general trend of clinch joints, as shown in Figure 10. According to the results shown in Figure 10, both heating temperatures of 100 °C and, 103 °C provide similar failure loads for clinch joints. However, the variation of failure loads for heating temperatures of 100 °C is a bit smaller than that for heating temperatures of, 103 °C probably due to the small cracks located near the necks shown in Figure 9b. Therefore, the heating temperatures of 100 °C is adopted in the following fatigue tests.

### 4.3. Die Depth Effects

**Figure 11:**
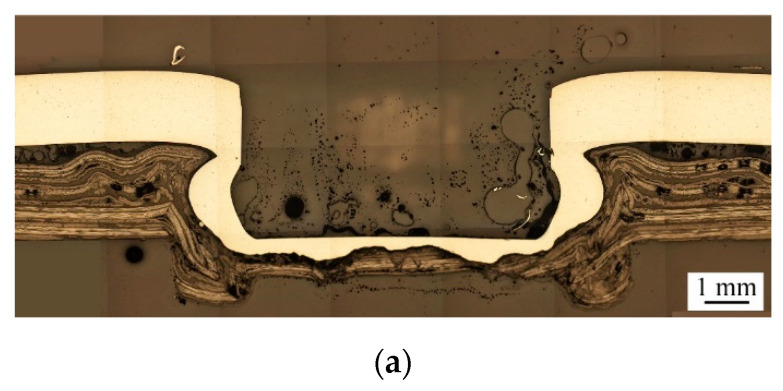

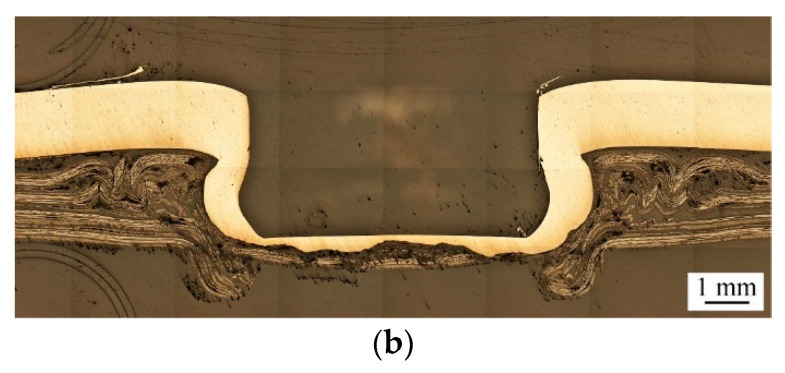
Cross-sectional micrographs of clinch joints made by (**a**) Die D1 ($d_{d}:0.7\;\mathrm{mm}$), and (**b**) Die D2 ($d_{d}:0.4\;\mathrm{mm}$). The Punch P1, punching force of 69 kN, heating temperature of 100 °C and Heating Mode B are used.

As discussed in the previous sections, excessive punching force or heating temperature may induce small or large cracks near the necks of clinch joints. These cracks can weaken their interlocks and corresponding joint strengths. Therefore, in this section, a new die, denoted as D2, with a shallower die depth of 0.4 mm is proposed to restrain the plastic deformation inside the clinch joint and eliminate the cracks near the necks as well. Figure 11 shows the effects of die depth on the geometry of clinch joints.

Two designs, Die D1 ($d_{d}:0.7\;\mathrm{mm}$) and Die D2 ($d_{d}:0.4\;\mathrm{mm}$), are considered. The clinch joints are made under a punching force of 69 kN, Heating Mode B, and a heating temperature of 100 °C with Punch P1. In Figure 11, Die D2 with shallower die depth gives the clinch joint a larger neck thickness and a smaller undercut size than Die D1 with deeper die depth. Also, nearly no cracks can be found near the necks shown in both Figure 11a,b. Note that the $d_{d}$ represents the depth of the die. This fact indicates that decreasing the die depth $d_{d}$ can restrain the plastic deformation inside the clinch joint and then weaken the interlock and corresponding joint strength.

**Figure 12:**
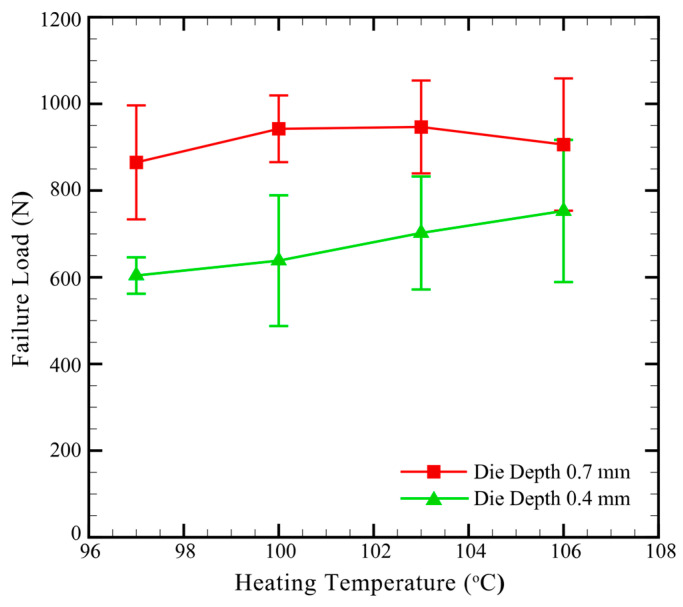
Failure load vs. heating temperature of clinch joints in CT specimens made by Die D1 ($d_{d}:0.7\;\mathrm{mm}$) and Die D2 ($d_{d}:0.4\;\mathrm{mm}$). The Punch P1, punching force of 69 kN, heating temperature of 100 °C and Heating Mode B are used.

Figure 12 plots the effects of die depth and heating temperature on the failure load of clinch joints in CT specimens.

In Figure 12, the failure loads of clinch joints made by Die D2 are smaller than those of clinch joints made by Die D1. As shown in Figure 11, Die D2 gives the clinch joints thicker necks and weaker interlocks than Die D1 under the same processing conditions. In Figure 12, the general trend of Die D2 is also different to that of Die D1. When the heating temperature increases, the failure load of Die D1 increases to the maximum at 103 °C and then decreases. As discussed in the previous section, increasing heating temperature improve the interlock but induce small and large surface cracks near the necks when the temperature is larger than 103 °C. The struggle between opposite effects results in the general trend of Die D1 shown in Figure 12. On the other hand, when the heating temperature increases, the failure load of Die D2 continuously and gradually increases. This fact indicates that the increasing heating temperature may improve the interlocks of clinch joints. Based on the discussions for Figure 11 and Figure 12, the clinch joints made by Die D1 still provides better interlock and larger failure load than those made by Die D2. Therefore, Die D1 is still adopted in the following fatigue tests.

### 4.4. Fatigue Test Results

**Figure 13:**
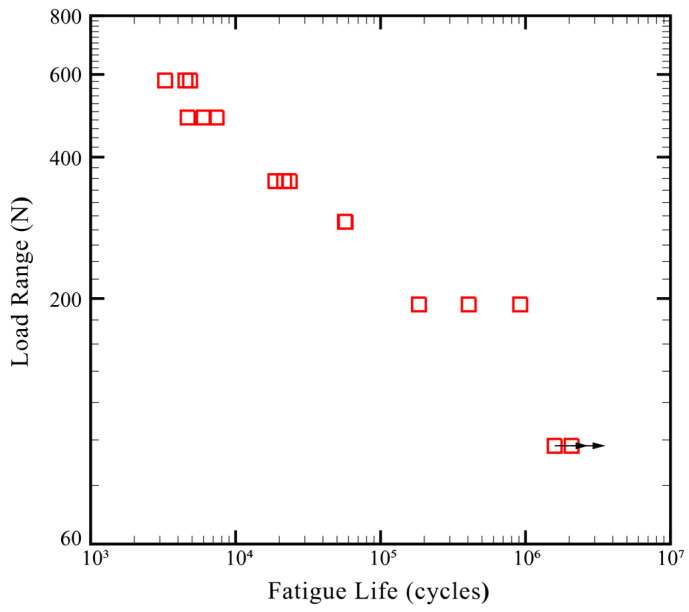
Fatigue test results of clinch joints in CT specimens made by Punch P1 and Die D1 under a punching force of 69 kN, Heat Mode B, and a heating temperature of 100 °C.

As discussed in the previous sections, an appropriate setup for CT specimens, including Punch P1, Die D1, Heating Mode B, a punching force of 69 kN, and a heating temperature of 100 °C, was obtained based on quasi-static tests. The clinch joints made under above processing parameters were then used in fatigue tests. Figure 13 plots the fatigue data (fatigue lives vs. load ranges) of clinch joints in CT specimens.

The load ranges (ΔF) were determined from 60%, 50%, 40%, 30%, 20%, and 10% failure load (883 N) in fatigue tests. The horizontal arrows here indicate that these fatigue tests were terminated manually since their fatigue lives exceeded 10^6^ cycles. In Figure 13, the fatigue life increases when the load range decreases. All the clinch joints were failed in the neck fracture failure mode based on experimental observations.

**Figure 14:**
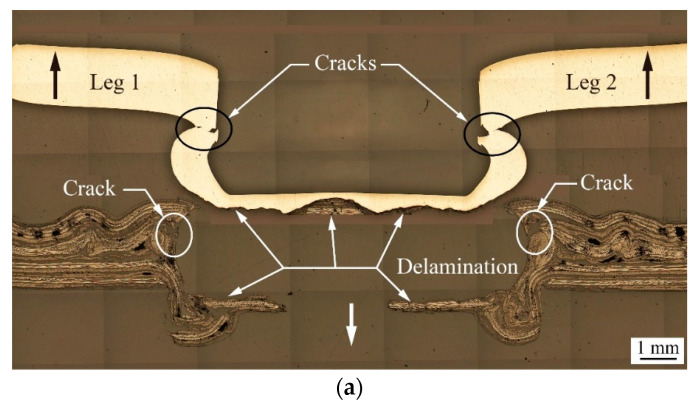

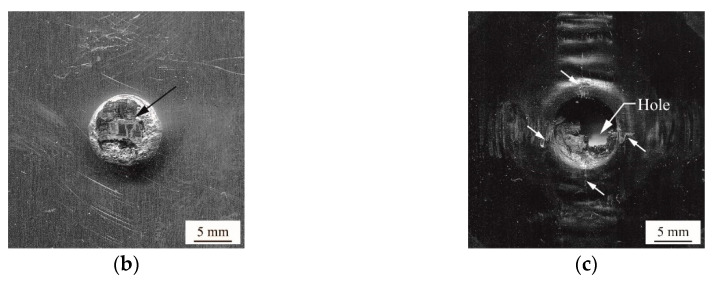
(**a**) A cross-sectional micrograph of a failed clinch joint subjected to quasi-static cross-tension loads. (**b**) The bottom surface of the upper sheet (AA5052) and (**c**) the top surface of the lower sheet (TP-CFRP) with a failed clinch joint.

Figure 14a shows the failure mechanism and failure mode of a clinch joint subjected to quasi-static cross-tension loads.

The left and right upward loads (thick black arrows) are applied to the upper sheet (AA5052). The front and back downward loads (thick white arrow) are applied to the lower sheet (TP-CFRP). Note that these two downward loads are overlapped from this viewpoint and therefore, only a single arrow can be seen. In Figure 14a, the inner button is partially cracked at necks (marked by arrows), while the outer button is partially cracked at the corners (marked by arrows) and delaminated at the bottom (marked by arrows). Once the cracks extend to certain level, the mechanical interlock of the clinch joint is failed, and then the inner button is pulled out of the outer button. The clinch joint is failed in the button separation failure mode. Noted that the CT specimens made under various processing conditions mentioned in the previous sections are all failed in this failure mode. Figure 14b,c are the bottom and top surfaces of the upper and lower sheets, respectively. In Figure 14b, the inner button (AA5052) has one piece of TP-CFRP on the bottom due to delamination shown in Figure 14a. In Figure 14c, the outer button (TP-CFRP) has a hole can be seen on the bottom due to the delamination shown in Figure 14a as well. Some portions of the corner of the outer button (TP-CFRP) are cracked, marked by small arrows, due to the cracks at the corners shown in Figure 14a. The fracture surfaces in Figure 14b,c correspond to the button separation failure mode.

**Figure 15:**
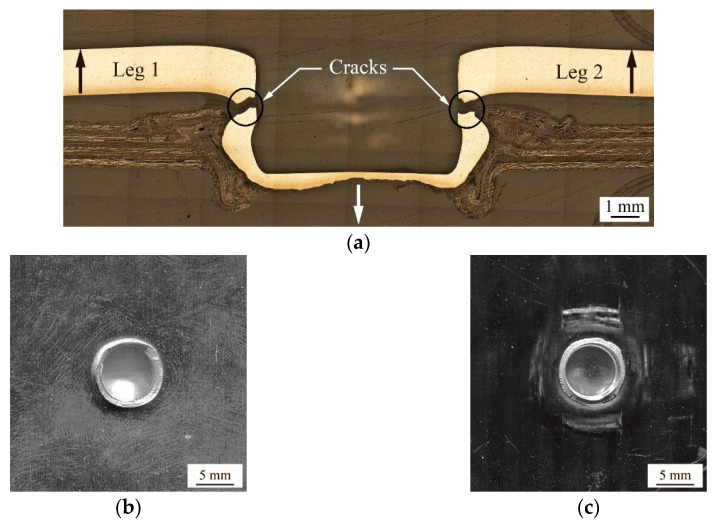
(**a**) A cross-sectional micrograph of a failed clinch joint subjected to cyclic cross-tension loads (ΔF=389 N). The fatigue life is 23,623 cycles. (**b**) The bottom surface of the upper sheet (AA5052) and (**c**) the top surface of the lower sheet (TP-CFRP) with a failed clinch joint.

Figure 15a shows the fatigue behavior and failure mode of a clinch joint subjected to cyclic cross-tension loads (ΔF=389 N).

The failed clinch joint has a fatigue life of 23,623 cycles. In Figure 15a, the left and right notches formed by the necks of the inner button become two cracks (marked by arrows) advancing towards the center horizontally. Once the necks of the inner button are fractured, the interlock between the inner and outer buttons is totally failed. The clinch joint is failed in the neck fracture failure mode. Figure 15b,c are the faying surfaces of the upper (AA5052) and lower (TP-CFRP) sheets, respectively. In Figure 15b, the inner button (AA5052) has a circular fracture surface on its neck. In Figure 15c, the outer button (TP-CFRP) holds the remaining portion of the inner button (AA5052) with a circular fracture surface. The fracture surfaces in Figure 15b,c correspond to the neck fracture failure mode.

## 5. Conclusions

This paper first studied effects of processing parameters on preheated (heat-assisted) clinching process to join AA5052 and TP-CFRP sheets for CT specimens. Four critical parameters, including punching force, die depth, heating mode, and heating temperature, were considered. Quasi-static tensile tests, fatigue tensile tests, and microscopic observations were conducted. Several important conclusions were obtained in the following:

- Under Heating Mode A (preheating TP-CFRP), the increasing punching force within this range has insignificant effects on the joint geometry and provides nearly constant failure loads. However, under Heating Mode B (preheating AA5052 and TP-CFRP), the increasing punching force has significant effects on the joint geometry and provides the best results among all at the punch force of 69 kN.
- The increasing heating temperature improves the interlock but also enlarges the cracks near the necks; therefore, the failure load increases and then decreases due to the competitions between these geometric features.
- The decreasing die depth weakens the interlock; therefore, the clinch joints made by shallow die have poor failure loads.
- An appropriate setup for CT specimens, including Heating Mode B, a punching force of 69 kN, a heating temperature of 100 °C, Punch P1, and Die D1, was obtained for fatigue tests. The average failure load was 883 N.
- In fatigue tests, the fatigue behavior, failure mode, and fatigue data of clinch joints in CT specimens were obtained.
- The clinch joints in quasi-static tests all failed in the button separation failure mode, while those in fatigue tests all failed in the neck fracture failure mode.

In the future, the appropriate setup of AA5052/TP-CFRP clinch joints obtained here can be used as a preliminary setup for production lines and manufacturing engineers. The failure load and fatigue performance of the appropriate setup can be used as basic mechanical properties for design engineers. The study of worst and best cases for the appropriate setup can be conducted by 25 or more replicates in order to have a normal distribution of the testing results for reliability analysis, as suggested by SAE AMS-W-6858A standard [40].

## Figures and Tables

**Table 1:** Mechanical properties of AA5052-H32 and TP-CFRP.

Material	Elastic Modulus (GPa)	Tensile Strength (MPa)	Elongation (%)	Density ($g/cm^{3}$)
AA5052-H32	70	193	12.0	2.68
TP-CFRP	250	4680	1.9	1.81

**Table 2:** Detailed dimensions of a circular punch and two circular dies.

Punch P1	$D_{p}$	$L_{p}$	$D_{s}$		
	7.0	6.25	16.0		
Die D1	$D_{d}$	$d_{d}$	$r_{g}$	$w_{g}$	$d_{g}$
	10.0	0.7	0.5	1.5	1.5
Die D2	$D_{d}$	$d_{d}$	$r_{g}$	$w_{g}$	$d_{g}$
	10.0	0.4	0.5	1.5	1.5
Unit: mm					

**Table 3:** A testing protocol of the quasi-static and fatigue tests.

Testing	Processes
Quasi-static Test	Prepare 3–5 CT specimens Conduct quasi-static tensile tests at rate of 5 mm/min Obtain average failure load
Fatigue Test	Prepare 5 CT specimens for each load range Determine load ranges from 60% to 10% of average failure load with load ratio R = 0.1 Conduct cyclic tensile tests at 2 Hz Obtain fatigue lives for each load range
